# Exploring Multifunctional Bioactive Components from *Podophyllum sinense* Using Multi-Target Ultrafiltration

**DOI:** 10.3389/fphar.2021.749189

**Published:** 2021-10-25

**Authors:** Huixia Feng, Guilin Chen, Yongli Zhang, Mingquan Guo

**Affiliations:** ^1^ Key Laboratory of Plant Germplasm Enhancement and Specialty Agriculture, Wuhan Botanical Garden, Chinese Academy of Sciences, Wuhan, China; ^2^ College of Life Sciences, University of Chinese Academy of Sciences, Beijing, China; ^3^ Sino-Africa Joint Research Center, Chinese Academy of Sciences, Wuhan, China; ^4^ Innovation Academy for Drug Discovery and Development, Chinese Academy of Sciences, Shanghai, China

**Keywords:** *Podophyllum sinense*, ultrafiltration-LC/MS, antiproliferative, anti-inflammatory, multi-target

## Abstract

*Podophyllum sinense* (*P. sinense*) has been used as a traditional herbal medicine for ages due to its extensive pharmaceutical activities, including antiproliferative, anti-inflammatory, antiviral, insecticidal effects, etc. Nevertheless, the specific bioactive constituents responsible for its antiproliferative, anti-inflammatory, and antiviral activities remain elusive, owing to its complicated and diversified chemical components. In order to explore these specific bioactive components and their potential interaction targets, affinity ultrafiltration with multiple drug targets coupled with high performance liquid chromatography/mass spectrometry (UF–HPLC/MS) strategy was developed to rapidly screen out and identify bioactive compounds against four well-known drug targets that are correlated to the application of *P. sinense* as a traditional medicine, namely, Topo I, Topo II, COX-2, and ACE2. As a result, 7, 10, 6, and 7 phytochemicals were screened out as the potential Topo I, Topo II, COX-2, and ACE2 ligands, respectively. Further confirmation of these potential bioactive components with antiproliferative and COX-2 inhibitory assays *in vitro* was also implemented. Herein, diphyllin and podophyllotoxin with higher EF values demonstrated higher inhibitory rates against A549 and HT-29 cells as compared with those of 5-FU and etoposide. The IC_50_ values of diphyllin were calculated at 6.46 ± 1.79 and 30.73 ± 0.56 μM on A549 and HT-29 cells, respectively. Moreover, diphyllin exhibited good COX-2 inhibitory activity with the IC_50_ value at 1.29 ± 0.14 μM, whereas indomethacin was 1.22 ± 0.08 μM. In addition, those representative constituents with good affinity on Topo I, Topo II, COX-2, or ACE2, such as diphyllin, podophyllotoxin, and diphyllin *O*-glucoside, were further validated with molecular docking analysis. Above all, the integrated method of UF–HPLC/MS with multiple drug targets rapidly singled out multi-target bioactive components and partly elucidated their action mechanisms regarding its multiple pharmacological effects from *P. sinense*, which could provide valuable information about its further development for the new multi-target drug discovery from natural medicines.

## Introduction

The majority of drugs introduced into the body exert pharmacological effects through interaction with various corresponding biological target molecules. For instance, enzymes which play key roles in the pathogenicity and progression of certain disease with specific physiological functions have exerted great potentials in drug discovery and development as drug targets (Yao et al., 2013; Zhu et al., 2013). Recent studies demonstrated that enzyme inhibitors can inhibit the activity of specific enzymes related to certain diseases, thereby possessing huge potentials for the development of therapeutic drugs (Rengasamy et al., 2014; Orhan, 2019). Natural products have long been evolved to exhibit a wide range of chemical and functional diversities (Zhang et al., 2020), and thus play a crucial role in the field of new drug development as valuable biological resources (Chang et al., 2016). On the one hand, however, the chemical components from natural products, such as medicinal plants, are complicated and often act in a multi-target manner; on the other hand, these phytochemicals bring varied biological activities but also pose huge challenges to screen and identify the specific bioactive compounds, and further elucidate their corresponding mechanisms of action (Chen et al., 2018). In this context, it is pivotal to develop an efficient strategy to correlate their complex chemical ingredients with diverse pharmacological activities in order to decipher the chemical basis of the drug effects. Traditional herbal medicine has been proposed to prevent or cure diseases in a multi-component and multi-target way, but little or no direct evidence was provided so far. In order to meet this tough challenge, the present work aims to develop an affinity ultrafiltration LC/MS based multi-drug target strategy by taking *Podophyllum sinense* as an example.

*P. sinense* (H.L. Li) (Christenh. & Byng) belongs to *Podophyllum*, Berberidaceae. It has been regarded as a traditional Chinese folk medicine for many years and is widely distributed in Hubei, Sichuan, and Shaanxi provinces (Terabayashi et al., 1984). As the medicinal part, its rhizomes have been traditionally used for the treatment of contusions, fractures, snake bites, traumatic swelling, tracheitis, rheumatic pain, etc. (Zhao et al., 2014). Phytochemical studies indicate that *P. sinense* contains multitudinous bioactive ingredients, such as lignans, flavonoids, anthraquinones, and volatile oils (Ma et al., 1993). Among these ingredients, lignans are reported as the primary bioactive components and demonstrate remarkable antiproliferative, anti-inflammatory, antiviral, insecticidal activities (Castro et al., 2003; Yuan et al., 2011; Liu et al., 2015). However, the specific bioactive compounds responsible for its antiproliferative, anti-inflammatory, and antiviral activities remain unknown till date.

The first step to solve this puzzle is to explore the phytochemical basis of the pharmacological effects of *P. sinense* for some specific drug targets that are used in order to elucidate the potential mechanism of action. In this way, it is very urgent to develop an effective and comprehensive approach to correlate its holistic bioactivity to its multi-purpose chemical components. Especially, the UF–LC/MS strategy could fill this gap, since it can simultaneously screen out and identify the bioactive components correlated to specific drug targets, which is especially applicable to the complex extracts of natural products (Zhao et al., 2009; Cieśla and Moaddel, 2016). Affinity ultrafiltration can facilitate the rapid separation of small molecule ligands bound with large molecular receptors (drug targets) from unbound molecules, and LC–MS can enable the quick identification of potential bioactive ligands after they are released from the targets (Qin et al., 2015). In this regard, the combination of the two techniques is not only vital to reveal the effective phytochemicals of natural products, such as medicinal plants, but also conducive to drug discovery (Qin et al., 2015; Ma et al., 2020). To date, UF–LC/MS technology has been successfully applied to screen bioactive ingredients in natural products by employing various disease-related drug targets (Mulabagal and Calderón, 2010; Lavecchia et al., 2013; Li et al., 2015; Chen et al., 2020; Zhang et al., 2021).

In light of these inspiring research ideas and the research progress in both phytochemicals and pharmacology on *P. sinense*, Topo I, Topo II, COX-2, and ACE2 were chosen as the potential drug targets to synchronously screen for their correlated bioactive components. Among these drug targets, DNA topoisomerase (Topo) catalyzes the DNA topological changes by breaking DNA strands transiently and plays a fundamental role in cell life (You and Gao, 2019). Generally, DNA topos are divided into type I and type II, therein Topo I fractures a single strand of duplex DNA to alter the topological structure, while Topo II breaks double strands to transform the topological structure (Delgado et al., 2018). These two enzymes, belonging to ribozymes, maintain the normal topological heterogeneity of the double-stranded DNA and are highly expressed in tumor cells (Liang et al., 2019). Consequently, they have become pivotal therapeutic targets for anticancer drugs currently, and their novel inhibitors are being actively sought (Sinha, 1995; Liang et al., 2019). With regard to cyclooxygenase 2 (COX-2), it is known as a key rate-limiting enzyme in the biosynthesis of the prostaglandins (PGs), which plays a dominant role in the pathophysiological process of chronic inflammation and cancer (Regulski et al., 2016; Mahboubi Rabbani and Zarghi, 2019). That is to say, the synthesis of PGs could be blocked by inhibiting the activity of COX-2, which further induces tumor cell apoptosis and inhibit tumor angiogenesis, invasion, and metastasis (Peng et al., 2018). Meanwhile, COX-2 inhibitors are a new generation of antitumor drugs with fewer side effects and high curative powers, which can be used as auxiliary drugs for the treatment of cancer, and their design and synthesis have been the hot spots for researchers (Mahboubi Rabbani and Zarghi, 2019). As for angiotensin-converting enzyme II (ACE2), it is a type I transmembrane metal carboxypeptidase, a homolog of carboxypeptidase ACE, which generates angiotensin II, the main active peptide of the renin–angiotensin system (RAS) (Hooper et al., 2020). ACE2 is widely expressed in the human lungs, heart, liver, kidney, and gastrointestinal mucosa (Leung et al., 2020). Meanwhile, ACE2 also plays an extremely critical role in the whole process of SARS-CoV-2 infection and disease (Shahid et al., 2020). The first point is that ACE2 can act as a receptor and bind to the viral S protein to mediate viral infection. Several studies have indicated that the viral infection could upregulate the ACE2 receptor, which is the main binding receptor used by the SARS-CoV-2 virus to enter host cells, and then exert an essential impact on the pathogenesis of severe lung failure (Gheblawi et al., 2020). Moreover, ACE2 can also participate in the process of acute lung injury and can be used as a key regulator to protect against acute lung injury (Kuba et al., 2010). In addition, ACE2, a powerful negative regulator, can regulate the RAS and participate in the process of lowering blood pressure (Garvin et al., 2020). Finally, as an essential biomolecule for the expression of neutral amino acid transporters on the surface of epithelial cells, ACE2 can bind to amino acid transporters and affect the process of amino acid absorption by the intestines and kidneys (Kuba et al., 2010). To the best of our knowledge, we, for the first time combine the UF–LC/MS method with the above-mentioned four drug targets that are closely correlated to the reported pharmacological effects of *P. sinense*, aiming to simultaneously explore the correlations between its bioactive chemical components and the underlying mechanisms of antitumor, anti-inflammation, and antivirus, which could provide the first piece of evidence to show that it acts in a multi-target and multi-component manner. In addition, this work would also provide a sufficient theoretical basis for further drug research and development efforts and rational clinical use of *P. sinense* in the near future.

## Materials and Methods

### Plant Materials

The medicinal material was the dried rhizome of *P. sinense*, and it was collected from the Shennongjia forest area in Hubei province. Afterward, the identification of the specimen was enthusiastically assisted by Professor Guangwan Hu, a senior taxonomist of the Key Laboratory of Plant Germplasm Enhancement and Specialty Agriculture (Wuhan Botanical Garden), Chinese Academy of Sciences. This material was packed in sealed polyethylene bags and then stored in the refrigerator at 4°C until further use.

### Chemicals and Reagents

The chemical standards of diphyllin, diphyllin *O*-glucoside, podophyllotoxin, kaempferol, kaempferol 3-*O*-glucoside, and quercetin 3-*O*-glucoside (Purity ≥98.0%) were bought from Chengdu Alfa Biotechnology Co., Ltd. (Chengdu, China), Shanghai Aladdin Bio-Chem Technology Co., Ltd. (Shanghai, China), and Shanghai Tauto Biotech Co., Ltd. (Shanghai, China), respectively. 5-Fluorouracil (5-FU), indomethacin, and etoposide were purchased from Shanghai Aladdin Bio-Chem Technology Co., Ltd. (Shanghai, China) and Shanghai Yuanye Bio-technology Co., Ltd. (Shanghai, China). Topo I, Topo II, COX-2, and ACE2 were provided by New England Biolabs (Ipswich, MA, United States), Sigma-Aldrich (St Louis, MO, United States), and Novoprotein (Shanghai, China), respectively. The protein-staining sulforhodamine B (SRB) cell proliferation and cytotoxicity detection kits were purchased from Shanghai BestBio Biotechnology Co., Ltd. (Shanghai, China). The COX-2 inhibitor screening assay kits were obtained from Shanghai Beyotime Biotechnology Co., Ltd. (Shanghai, China). The 30 kDa cut-off centrifugal ultrafiltration filters (YM-30) were bought from Millipore Co., Ltd. (Bedford, MA, United States). The Millipore membranes (0.22 µm) were purchased from Tianjin Jinteng Experiment Equipment Co., Ltd. (Tianjin, China). The HPLC grade solvents of formic acid (FA) and acetonitrile (ACN) were purchased from TEDIA Company Inc. (Fairfield, OH, United States). The pure water for the HPLC–ESI–MS was prepared by the EPED water system (Yeap Esselte Tech. Co., Nanjing, China). All other analytical grade chemicals and solvents were obtained from Shanghai Chemical Reagent Corp. (Shanghai, China).

### Preparation of the Extracts from *P. sinense*

For the sample preparation, the rhizome of *P. sinense* was first crushed with a high-speed disintegrator. An aliquot of 200 g raw powders was weighed accurately and then was soaked in 90% ethanol overnight with the material-to-liquid ratio of 1:10. Next, ultrasonic extraction was performed at room temperature for 30 min. Then, the filtrate was obtained by vacuum filtration. And, the above extraction process was repeated two times. Finally, the three filtrates were combined and concentrated under reduced pressure to obtain ethanol extracts of *P. sinense*.

### Affinity Ultrafiltration Procedures

The present affinity ultrafiltration screening procedures were carried out based on our previous methods with slight modifications (Chen et al., 2020). In brief, the crude extracts of *P. sinense* were accurately weighed and fully dissolved in tri (hydroxymethyl) aminomethane hydrochloride (Tris-HCl, pH 7.80) or phosphate buffer saline (PBS, pH 6.87) to prepare the sample solutions for ultrafiltration screening. One hundred microliters of tested sample solution (at a final concentration of 2 mg/ml) was incubated with 10 µl of Topo I (5 U), Topo II (2 U), COX-2 (4 U), or ACE2 (0.5 µg) in a constant temperature shaking incubator at 37°C for 40 min. After incubation, the mixtures were transferred to a 30 KD ultrafiltration tube and then centrifuged at 10,000 rpm for 10 min. Then, the filtrates were eluted by 200 μl of Tris-HCl or PBS three times to eliminate unbound ligands. Subsequently, the ligands specifically bound to Topo I, Topo II, COX-2, or ACE2 were released from the complexes by cultivating 10 min at room temperature with 200 µl of 90% methanol, and after that they were centrifuged at 10,000 rpm for 10 min with three times. Ultimately, all the residues above were collected and reestablished in 50 µl of methanol after lyophilizing, and then analyzed using the HPLC–ESI–MS/MS. Meanwhile, the inactivated enzyme solution, which was placed in boiling water for 10 min, was regarded as the control group adopting the same procedure as above.

### HPLC–ESI–MS/MS Conditions

The crude extracts of *P. sinense* were dissolved in methanol ahead of schedule. The two filtrates mentioned above were analyzed straight away employing the HPLC–ESI–MS/MS system with a TSQ Quantum Access MAX mass spectrometer (Thermo Fisher Scientific, San Jose, CA, United States), which is coupled to a Thermo Access 600 HPLC system. Chromatographic separations were performed on a Waters Symmetry RP-C18 column (4.6 mm × 250 mm, 5 µm) and connected with a guard column at 30°C. The mobile phase consisted of 0.1% FA-H_2_O (A) and ACN (B), and the optimized gradient chromatographic conditions were carried out as follows: 0 min, 5% (B); 40 min, 95% (B). The injection volume was adjusted to 10 μl, the flow rate was set as 0.8 ml/min, and the UV chromatograms were monitored at a wavelength of 292 nm.

Considering the ESI-MS/MS analysis, the triple quadrupole mass spectrometer electrospray ionization (ESI) was adopted to obtain various fragment ions in the positive ion mode. The optimized instrument conditions were implemented as follows: the spray voltage, vaporizer temperature and capillary temperature were 3,000 V, 350 and 250°C, respectively; the cone voltage, sheath gas pressure, and aux gas pressure were 40.0 V, 40, and 10 psi, respectively; the acquisition data of *m/z* ranging from 150 to 1,000 were gained in the full-scan mode; the collision energy varied from 30 to 45 eV for the MS/MS analysis in the data-dependent scanning mode. Eventually, all the above data available were acquired from the professional software of Thermo Xcalibur ChemStation (Thermo Fisher Scientific).

### Anti-Proliferative Assay *in vitro*

Cell proliferation was determined by SRB assay according to the manufacturer’s instructions (Vichai and Kirtikara, 2006). Non-small lung cell cancer (A549) and colon cancer (HT-29) cell lines were maintained in Dulbecco’s Modified Eagle’s Medium (DMEM) replenished with 1% penicillin–streptomycin and 10% fetal bovine serum (FBS), and further incubated in 5% CO_2_ and 90% relative humidity at 37°C. In brief, these two cell lines were seeded into 96-well plates with a density of 1.0 × 10^4^ per well and grown overnight. Next, the cells were treated with various concentrations of samples and incubated for 48 h. The medium with less than 0.1% DMSO was used as the blank control in this assay, and 5-FU as well as etoposide was regarded as the positive controls. The optical density (OD) value of each well was recorded at 540 nm with a multifunctional microplate reader (Tecan Infinite M200 PRO, TECAN, Männedorf, Switzerland). The inhibition rate was estimated and calculated, as shown in the formula:

$$\mathrm{Inhibition}\;\mathrm{rate}\;\left(\%\right)\;=\;\frac{\left(OD_{C}-OD_{S}\right)}{OD_{C}}\;\times \;100\%,$$

where OD_C_ is the absorbance value of the blank control, and OD_S_ is the absorbance value of the tested sample or positive control. The data were expressed as means ± standard deviation (SD) of three replicates.

### COX-2 Inhibitory Assay *in vitro*

The COX-2 inhibitory assay was performed utilizing a COX-2 inhibitor screening kit to detect the *in vitro* anti-inflammatory activity of these screened compounds as previously described, with slight modifications (Fan et al., 2015). In brief, COX-2 probe (50 X), COX-2 cofactor (50 X), and COX-2 substrate (50 X) were placed in DMSO at 37°C for 2 min to promote melting, and all the above solutions were diluted 10 times with the COX-2 assay buffer. Next, Tris-HCl (150 μl, pH 7.90), COX-2 cofactor (10 μl), and COX-2 (10 μl) solutions were mixed in a 96-well blackboard. An aliquot of 10 μl sample solution with the tested concentrations of 0.625–20 μM was added into mixtures and incubated at 37°C for 10 min. Subsequently, 10 μl of COX-2 probe was added to each well, and the reaction mixture was initiated by adding 10 μl of COX-2 substrate quickly. Finally, fluorescence measurement was performed after incubation in the dark at 37°C for 5–20 min. The excitation wavelength of the reaction mixtures was set at 560 nm, and the emission wavelength was monitored at 590 nm. The calculation of inhibition rate using the formula was as follows:

$$\mathrm{Inhibition}\;\mathrm{rate}\;\left(\%\right)\;=\;\frac{\left(\mathrm{RF}U_{1}-\mathrm{RF}U_{2}\right)}{\left(\mathrm{RF}U_{3}-\mathrm{RF}U_{4}\right)}\;\times \;100\%.$$

Here, RFU_1_, RFU_2_, and RFU_3_ represented the relative fluorescence unit of 100% enzyme activity control group, tested sample, and the blank control group, respectively. Meanwhile, indomethacin, a kind of non-steroidal anti-inflammatory drug (NSAID) (Lucas, 2016), was regarded as the positive control. COX-2 assay buffer without COX-2 and the tested sample were served as the blank control. Each sample solution was tested in three parallels, and the final experimental results were expressed in the form of means ± SD.

### Molecular Docking Analysis

Molecular docking assay was applied to simulate and analyze the binding energy, binding site, and the mode of action between screened potential phytochemicals and Topo I, Topo II, COX-2 or ACE2 by employing the computational program AutoDock Vina 1.5.6 and Discovery Studio 4.5 Client (Towler et al., 2004). The analytical procedures were primarily comprised of the determination of the interaction sites between the receptor and the ligand, generation of the scoring system, and the calculation of docking results. Firstly, the 3D structure of Topo I (PDB ID: 1T8I), Topo II (PDB ID: 3QX3), COX-2 (PDB ID: 1CX2), and ACE2 (PDB ID: 1R42) was downloaded from the PDB databases (www.rcsb.org). The structure of the ligand was processed by ChemBioDraw Ultra 14.0, and the file was modified to PDB format. After that, the receptor and ligand were processed by hydrogenating, calculating the charge, and confirming the protonation state using AutodockTools software. The receptor and the ligand were converted to readable file format (pdbqt). Subsequently, AutoGrid processing was performed to set the box size (60 × 60 × 60), spacing (0.375), and the docking active site of Topo I (*X*: 21.474; *Y*: −2.226; *Z*: 27.863), Topo II (*X*: 33.026; *Y*: 95.765; *Z*: 51.567), COX-2 (*X*: 24.263; *Y*: 21.528; *Z*: 16.497), or ACE2 (*X*: 52.874; *Y*: 68.399; *Z*: 33.501). Finally, after setting the relevant parameters, AutoDock calculations on the genetic algorithm (run 50 times) were conducted for the structure of small molecule receptors in a flexible way. In addition, 5-FU and camptothecin were considered as the positive control against Topo I. MLN-4760 and chloroquine were regarded as the positive control against ACE2. Etoposide and indomethacin were deeded as the positive control against Topo II and COX-2, respectively.

### Statistical Analysis

All the resulted values were expressed as means ± SD in triplicate, and the differences were regarded to be significant at *p* < 0.05 for all analyses. The half maximal inhibitory concentration (IC_50_) values were obtained by means of plotting the inhibitory rates against tested sample concentrations with a series of different concentration gradients. Statistical data analysis in the present study was performed by applying the GraphPad Prism 8 Software (GraphPad Software Corp., Sam Diego, CA, United States), the Origin 2019 Software (OriginLab Corp., Northampton, MA, United States), the ChemBioOffice 14.0 Software (CambridgeSoft Corp., Cambridge, MA, United States), and the SPSS 16.0 Software (SPSS Inc., Chicago, IL, United States).

## Results

### Ultrafiltration Analysis of Topo I, Topo II, COX-2, and ACE2 Ligands in *P. sinense*

**FIGURE 1:**
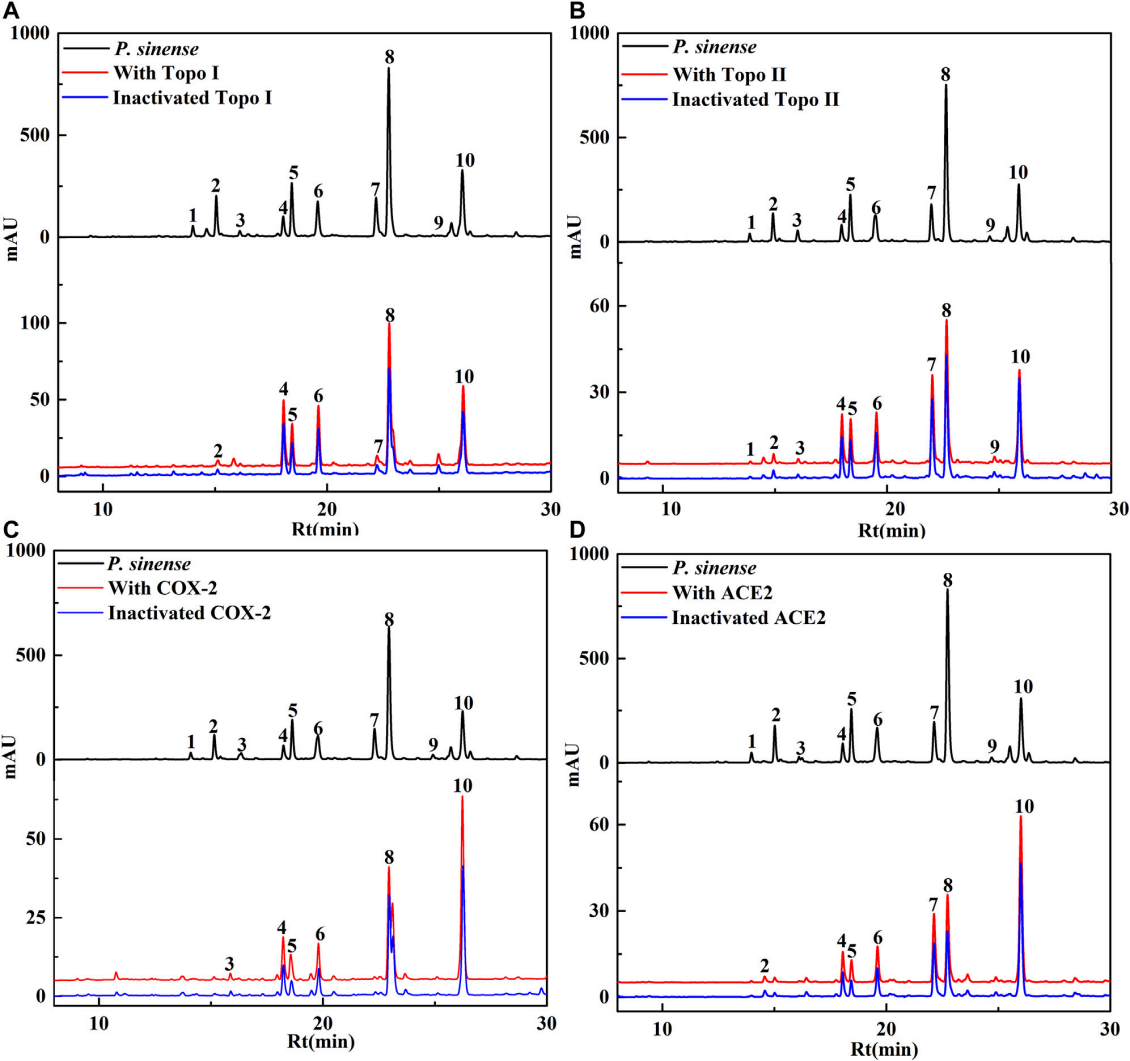
HPLC chromatograms of the chemical constituents in *P. sinense* were obtained after ultrafiltration at 292 nm. The black solid line represents HPLC profiles of the crude extracts of *P. sinense* without ultrafiltration; the red line and blue line represent the total extract of *P. sinense* with activated and inactivated Topo I **(A)**, Topo II **(B)**, COX-2 **(C)**, and ACE2 **(D)**, respectively.

*P. sinense* has been utilized as a folk medicine for centuries in China. Numerous studies have shown that *P. sinense* possesses significant antiproliferative, anti-inflammatory, and antiviral activities (Castro et al., 2003; Yousefzadi et al., 2010). In order to further offer a new theoretical basis for the clinical use of the *P. sinense*, this study took advantage of the UF–LC/MS strategy with four drug targets (Topo I, Topo II, COX-2, and ACE2) to rapidly screen out and identify active constituents corresponding to its antiproliferative, anti-inflammatory, and antiviral activities as reported. After incubation with Topo I, Topo II, COX-2 or ACE2 for affinity ultrafiltration, the bound ligands in *P. sinense* were released and further characterized by HPLC–MS analysis. Figure 1 displayed that the HPLC chromatograms of the chemical components from *P. sinense* exhibited obvious differences after the interaction with Topo I, Topo II, COX-2, and ACE2, respectively.

Based on the variations in the chromatographic peaks of the components from the crude extracts of *P. sinense* before and after incubated with Topo I, Topo II, COX-2, and ACE2, the enrichment factor (EF) values will be applied to evaluate the degree of affinities between the potential bioactive components from *P. sinense* and the target enzymes. The EF value was computed, as shown in the following formula:

$$\mathrm{EF}\;\left(\%\right)\;=\;\frac{\left(A_{1}-A_{2}\right)}{A_{0}}\;\times \;100\%.$$

Here, A_1_, A_2_, and A_0_ represent the peak area of each chromatographic peak from the crude extracts of *P. sinense* that is treated with activated, inactivated, and without Topo I, Topo II, COX-2, and ACE2, respectively (Chen et al., 2020). When employed to evaluate the affinity between the active constituents and the enzyme, the EF value implies the ability of diverse components to bind to the target enzyme. These components were further identified based on the MS/MS data in comparison with the spectra of corresponding standards or related references, and the results were revealed in Table 1. As expected, the EF values of each constituent listed in the table deviated from each other. It was observed that 7, 10, 6, and 7 chemical components in the HPLC chromatograms incubated with active Topo I, Topo II, COX-2, and ACE2 in *P. sinense* were provided with higher peak areas than that of the inactivated control group, respectively. The results indicated these constituents exerted specific binding towards Topo I, Topo II, COX-2, or ACE2, and thus were considered as primary potential ligands for Topo I, Topo II, COX-2, and ACE2, respectively. Notably, it is the first time to clearly describe the primary Topo I, Topo II, COX-2, and ACE2 ligands in *P. sinense*.

**TABLE 1:** EFs and the UF–LC/MS data of the bioactive compounds bound to Topo I, Topo II, COX-2, and ACE2 from the crude extracts of *P. sinense*.

Peak No.	Rt^a^ (min)	EFs^b^ (%)				[M+H]^+^	Characteristic fragment (*m/z*)	Identification
		Topo I	Topo II	COX-2	ACE2			
1	13.88	—	0.20	—	—	465	465, 303, 85	Quercetin 3-*O*-glucoside^c^
2	14.92	0.37	0.18	—	0.22	449	449, 287	Kaempferol 3-*O*-glucoside^c^
3	16.02	—	0.49	1.49	—	415	313, 298, 282, 229	Podophyllotoxin isomer^d^
4	17.98	9.36	2.86	4.40	2.76	543	381, 363, 351, 333, 305	Cleistanthin B isomer^d^
5	18.37	2.38	1.15	1.39	0.65	397	397, 313, 282, 229	*β*-Apopicropodophyllin^d^
6	19.49	3.07	0.90	1.14	1.37	543	381, 363, 351, 333, 305	Diphyllin *O*-glucoside^c^
7	21.98	0.48	1.04	—	3.10	287	287, 241, 213, 165, 153	Kaempferol^c^
8	22.64	3.89	1.17	1.61	1.31	415	397, 313, 282, 247, 229	Podophyllotoxin^c^
9	24.59	—	1.08	—	—	395	395, 380, 351, 336, 320	Chinensinaphthol methyl ether^d^
10	25.88	4.41	0.26	5.25	3.68	381	381, 363, 351, 333, 305	Diphyllin^c^

a Rt, retention time.

b EFs, enrichment factors.

c Compared with the corresponding standards.

d Identified based on the published literature.

### Structural Identification of Topo I, Topo II, COX-2, and ACE2 Ligands in *P. sinense*

**FIGURE 2:**
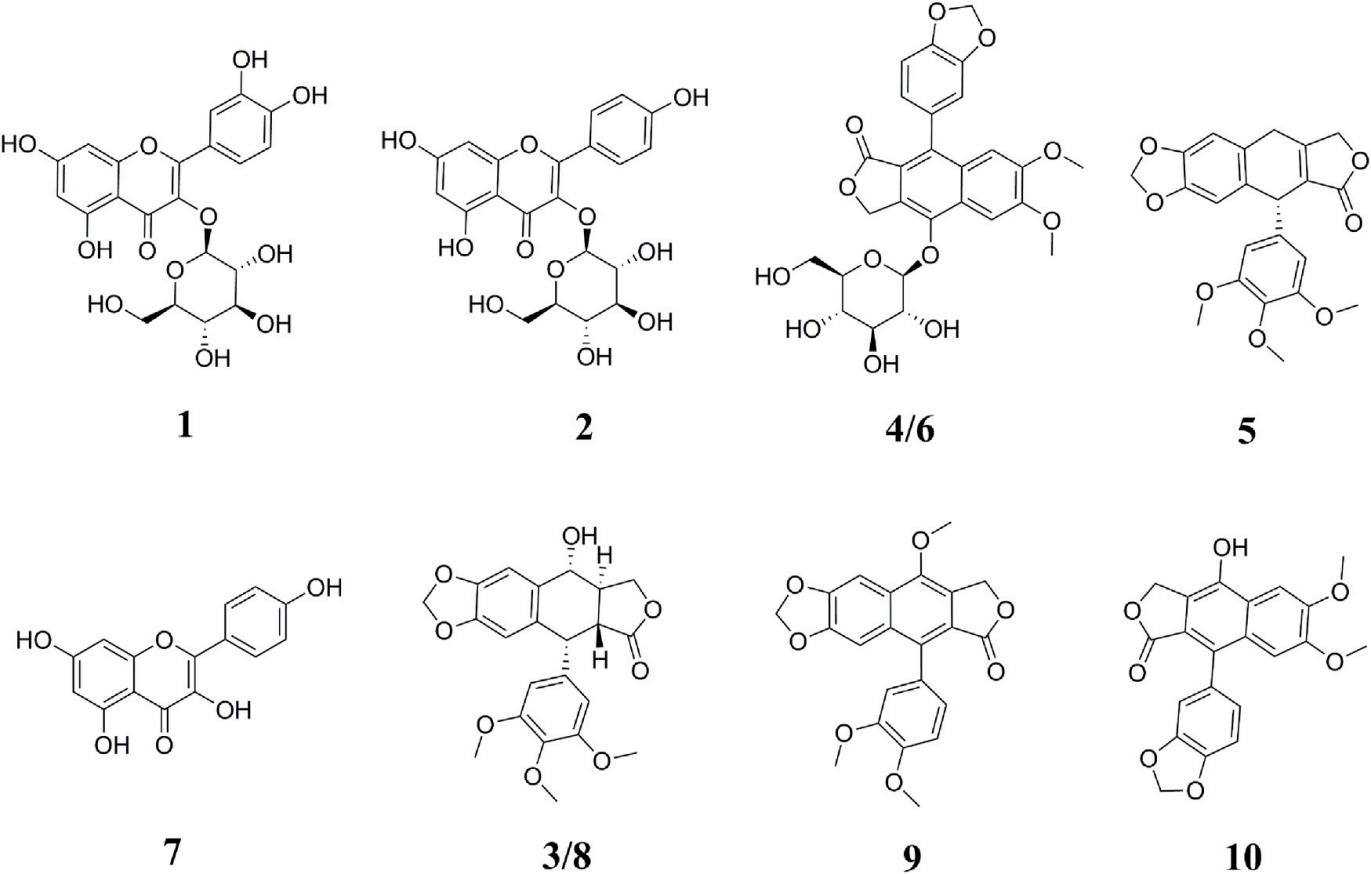
Chemical structures of bioactive compounds identified from *P. sinense* based on the MS/MS spectra or the corresponding standards. The peak numbers in this figure correspond to those listed in Table 1.

The samples after affinity ultrafiltration screening were subsequently analyzed by HPLC–ESI–MS/MS to exploit the ligands corresponding to Topo I, Topo II, COX-2, and ACE2, respectively. The HPLC–ESI–MS/MS analysis was carried out in the positive ion mode. The retention time (Rt), quasi-molecular ([M+H]^+^), and the characteristic MS/MS fragments of each component were shown in Table 1. By comparing with the MS/MS fragments of the chemical composition reported in the literature, as well as the spectrum of the corresponding standards, 10 potential bioactive compounds screened from the crude extracts of *P. sinense* were identified in total, and their chemical structures were presented in Figure 2.

**FIGURE 3:**
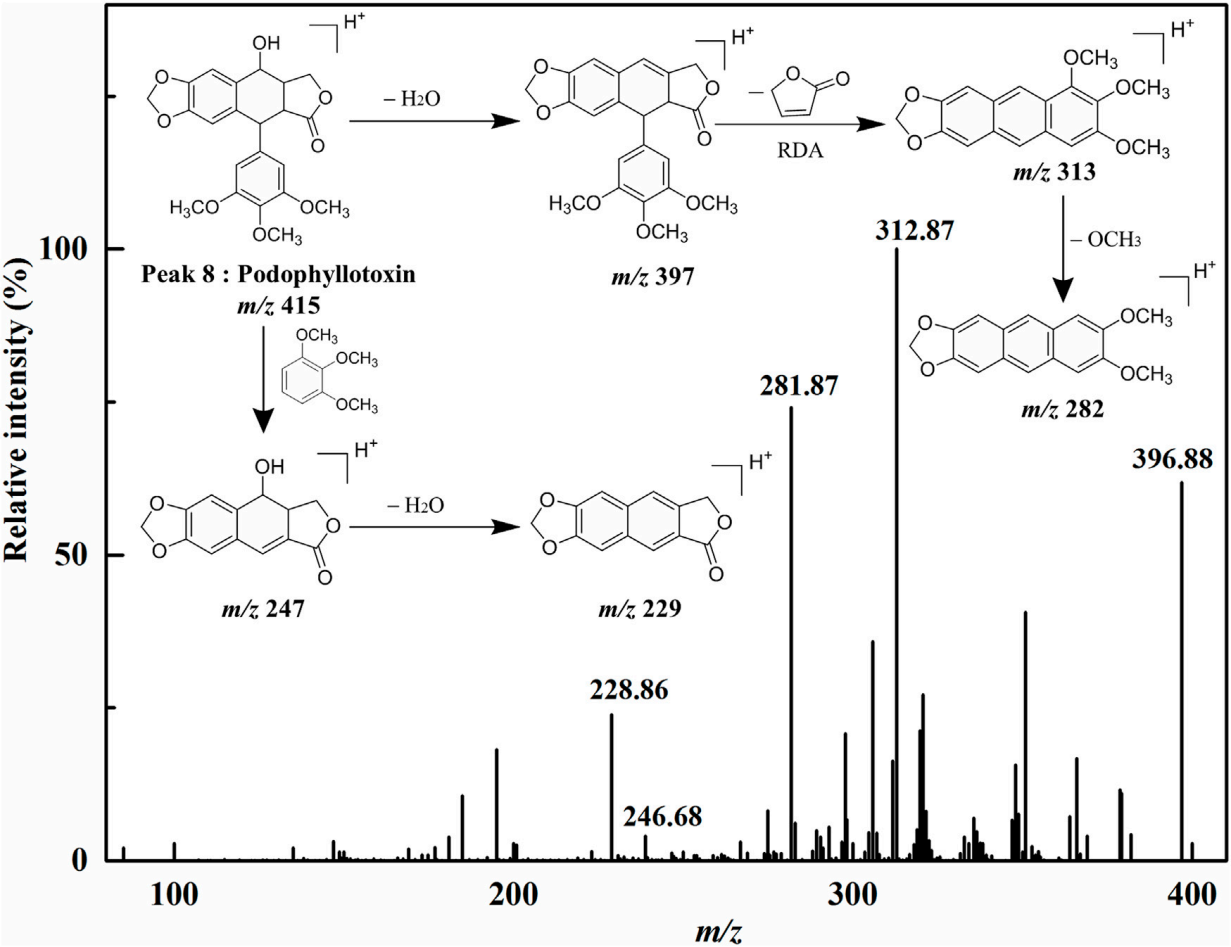
ESI-MS/MS spectra and the proposed fragmentation pathways of Peak 8.

Peak 1 exhibited the [M+H]^+^ ion at *m/z* 465, and the aglycon ion [M+H-C_6_H_10_O_5_]^+^ was further generated at *m/z* 303 by losing a hexose moiety, which was speculated to be quercetin monoglycoside. By comparison with the ion fragments of the existing standards, thus the peak 1 was identified as quercetin 3-*O*-glucoside (isoquercitrin, calculated for C_21_H_20_O_12_, 464 Da). Peak 2 presented the [M+H]^+^ ion at *m/z* 449, and the aglycone fragment ions at *m/z* 287 indicated the loss of a hexose moiety [M+H-C_6_H_10_O_5_]^+^. Comparing the retention time and the MS/MS spectra with the ion fragments of the corresponding standards, peak 2 was determined to be kaempferol 3-*O*-glucoside (astragalin, calculated for C_21_H_20_O_11_, 448 Da). Peak 7 ([M+H]^+^ at *m/z* 287) was considered to be kaempferol (calculated for C_15_H_10_O_6_, 286 Da) by comparing its LC–MS/MS with the standard compound. Peak 8 showed the [M+H]^+^ ion at *m/z* 415 and further suffered a neutral loss of the water molecular moiety to produce the fragment ion of the [M+H-H_2_O]^+^ ion at *m/z* 397. Typically, the ions at *m/z* 313 and *m/z* 282 were accorded with the characteristic retro Diels–Alder (RDA) cleavage ([M+H-H_2_O-C_4_H_4_O_2_]^+^) and the neutral loss of the methomyl moiety ([M+H-H_2_O-C_4_H_4_O_2_-OCH_3_]^+^), respectively. In addition, the fragment ions of [M+H-C_6_H_3_(OCH_3_)_3_]^+^ at *m/z* 247 and [M+H-C_6_H_3_(OCH_3_)_3_-H_2_O]^+^ at *m/z* 229 were regarded as the successive neutral losses of the trimethoxyphenyl moiety and the water molecular moiety, respectively. Based on the comparison of the LC–MS/MS analysis to the corresponding standards, peak 8 was thus identified as podophyllotoxin (calculated for C_22_H_22_O_8_, 414 Da). Similarly, peak 3 ([M+H]^+^ at *m/z* 415) and peak 5 ([M+H]^+^ at *m/z* 397) generated the parallel characteristic fragment ions in contrast to peak 8 (Lautié et al., 2008). Hence, peak 3 was tentatively identified as the isomers of peak 8. As for peak 5, it was tentatively suggested as *β*-apopicropodophyllin (calculated for C_22_H_20_O_7_, 396 Da) when compared with those of the ESI–MS/MS spectra and the corresponding fragmentation pathways. For the sake of further elucidating the structures of those lignans, the ESI–MS/MS spectra and representative fragmentation pathways of Peak 8 were revealed in Figure 3. Tentatively, comparing their MS/MS spectra with the corresponding standards and the related literature reported previously, peaks 6 and 10 were deduced as diphyllin *O*-glucoside (cleistanthin B, calculated for C_27_H_26_O_12_, 542 Da) and diphyllin (calculated for C_21_H_16_O_7_, 380 Da) (Jullian-Pawlicki et al., 2015), respectively. Peaks 4 and 6 displayed the identical [M+H]^+^ ion at *m/z* 543 and the MS/MS spectra at *m/z* 381, 363, 351, 333, and 305, implying that they were structural isomers. Fragment ions at *m/z* 381, 363 and 333 were obtained by the losses of a hexose moiety, the methyl moiety and CO_2_ moiety, respectively. For Peak 9 ([M+H]^+^ at *m/z* 395), fragment ions of [M+H-CH_3_]^+^ at *m/z* 380 and [M+H-CO_2_]^+^ at *m/z* 351 were produced by the losses of the methyl moiety and CO_2_ moiety, respectively. Based on the comparison of the conceivable fragmentation pathways and the MS/MS spectra of the published literature, peak 9 was deduced as chinensinaphthol methyl ether (calculated for C_22_H_18_O_7_, 394 Da) (Qin et al., 2016).

### Anti-Proliferative and COX-2 Inhibitory Assays on Ligands Screened from *P. sinense*

**FIGURE 4:**
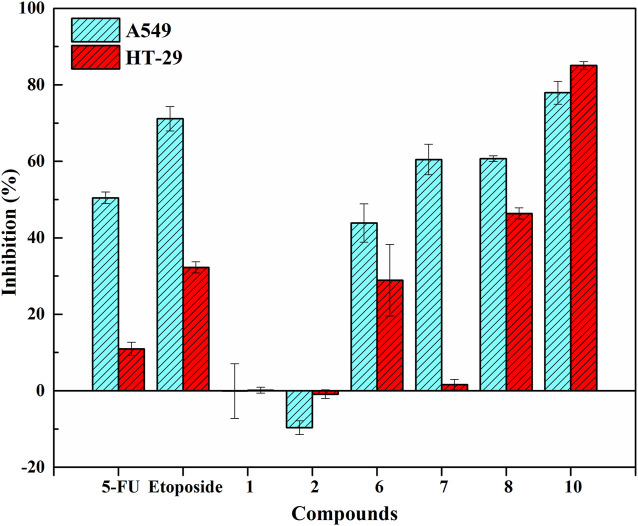
Inhibitory effects of these selected components and two positive drugs on A549 and HT-29 cells.

In order to associate pharmacological effects with potential active phytochemicals, antiproliferative and COX-2 inhibitory assays *in vitro* were performed to assess and verify the inhibitory effects of several screened ligands on Topo and COX-2. Herein, 5-FU and etoposide were considered as the positive controls for antiproliferative assay *in vitro*. Indomethacin was used as the positive control for COX-2 inhibitory assays *in vitro*. For antiproliferative assay *in vitro*, Figure 4 showed the inhibitory effects of these selected components and two positive drugs on A549 and HT-29 cells. Among these compounds, diphyllin (Peak 10) and podophyllotoxin (Peak 8) exhibited higher inhibitory rates against A549 and HT-29 cells at a concentration of 100 μM in contrast to 5-FU and etoposide, thereinto, the IC_50_ values of diphyllin were computed at 6.46 ± 1.79 and 30.73 ± 0.56 μM on A549 and HT-29 cells, respectively. As for COX-2 inhibitory assays *in vitro*, Table 2 enumerated the IC_50_ values of the representative compounds screened from *P. sinense*. Diphyllin, as the representative potentially anti-inflammatory component with the highest EF value, displayed good inhibition with the IC_50_ value at 1.29 ± 0.14 μM, compared to indomethacin at 1.22 ± 0.08 μM. Furthermore, podophyllotoxin and diphyllin *O*-glucoside (Peak 6) with the lower EF values exhibited relatively low inhibition with the IC_50_ values at 10.49 ± 0.61 μM and greater than 20 μM in comparison to diphyllin and indomethacin, respectively. Consequently, it was worthwhile to screen out and identify these potentially active components from *P. sinense*.

**TABLE 2:** The IC_50_ values of representative compounds screened from *P. sinense* against COX-2 *in vitro*.

No.	Compounds	IC_50_ (μM)^a^
1	Diphyllin	1.29 ± 0.14
2	Podophyllotoxin	10.49 ± 0.61
3	Diphyllin *O*-glucoside	>20
4	Indomethacin^b^	1.22 ± 0.08

a IC_50_, half-maximal inhibitory concentrations.

b Positive control.

### Molecular Docking Analysis

**FIGURE 5:**
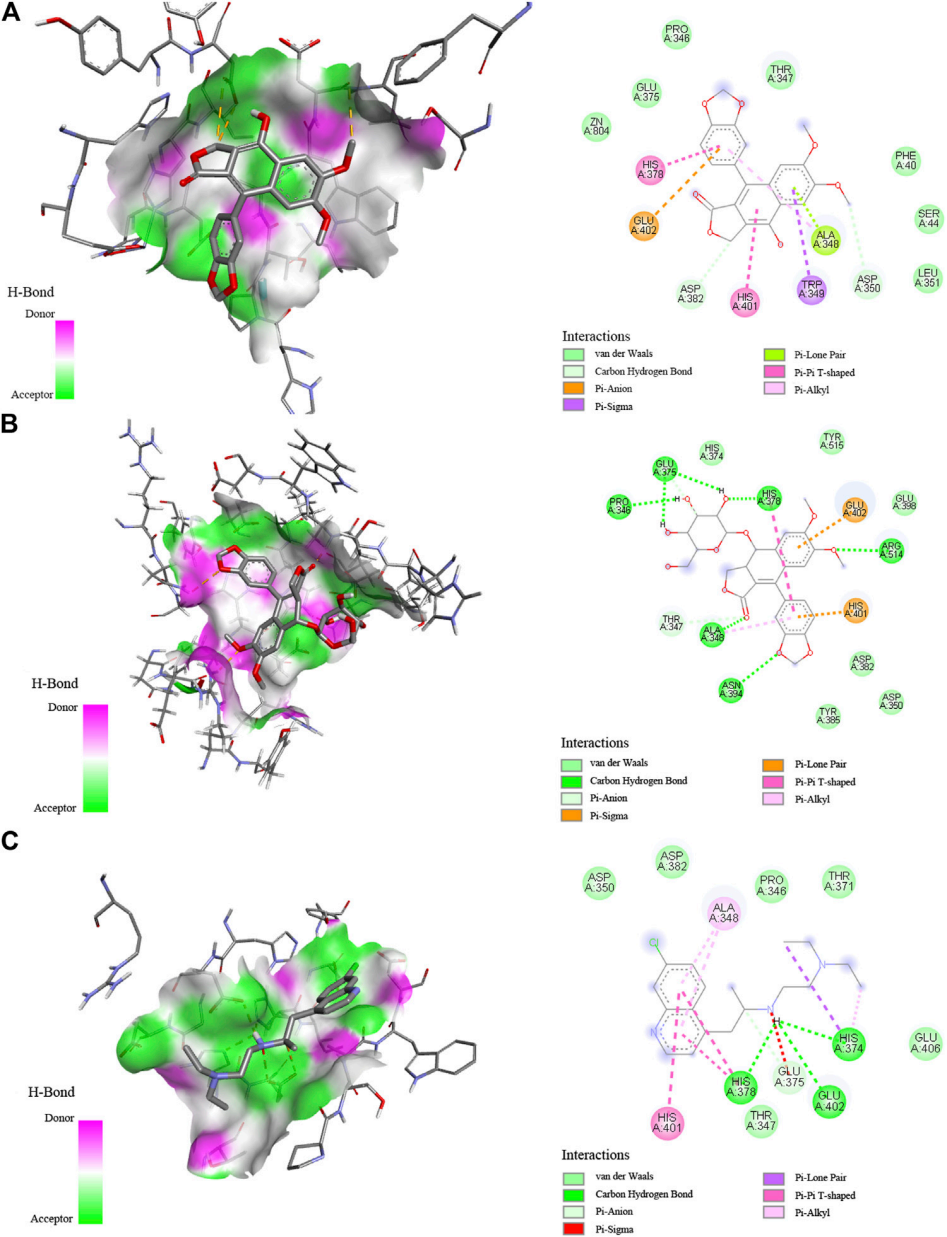
Interaction between ACE2 and diphyllin **(A)**, diphyllin *O*-glucoside **(B)**, or chloroquine **(C)** by molecular docking analysis.

Molecular docking is a crucial approach to predict and study the interactions between receptors and ligands. In order to further explore and comprehend the possible mechanism of action, molecular docking studies were carried out to simulate the interactions between Topo I, Topo II, COX-2, and ACE2 as well as several bioactive compounds screened from *P. sinense*. Table 3 displayed the molecular docking results of potential bioactive ligands screened from *P. sinense* and positive drugs against Topo I, Topo II, COX-2, and ACE2. Therein, the results of the 2D and 3D ligand-to-target interactions between ACE2 and diphyllin, diphyllin *O*-glucoside or chloroquine were shown in Figure 5.

**TABLE 3:** The molecular docking results of potential bioactive ligands screened from *P. sinense* and positive drugs against Topo I, Topo II, COX-2 and ACE2.

No.	Compounds	Drug targets	BE^a^ (kcal/mol)	IC_50_ (μM)	H-bond atoms^b^
1	Diphyllin	Topo I	−6.72	11.95	Da113, Dc112, Asp533, Arg364
		COX-2	−9.95	0.05	Ala527, Arg120, His90, and Tyr355
		ACE2	−7.07	6.52	Asp350, Asp382
2	Diphyllin *O*-glucoside	COX-2	−4.43	568.94	Arg120, Gly526, Ser530, Tyr355, Tyr385
		ACE2	−6.59	14.87	Ala348, Arg514, Asn394, His378, Glu375, Pro346
3	Podophyllotoxin	Topo II	−9.45	0.12	Da12, Dt9, Gln778
4	Kaempferol 3-*O*-glucoside	Topo I	−3.57	2,350.00	Arg364, Arg488, Dg12, Thr718
		Topo II	−5.28	134.95	Dt9, Arg503
5	Camptothecin^c^	Topo I	−7.57	2.85	Da113, Dc112, Glu356, Lys425
6	5-FU^d^	Topo I	−3.70	1,950.00	Met428, Tyr426
7	Etoposide^e^	Topo II	−7.62	2.59	Da12, Gln778
8	Indomethacin^f^	COX-2	−9.18	0.19	Glu524
9	MLN-4760^g^	ACE2	−4.27	738.62	Glu375, Glu402, Thr371
10	Chloroquine^h^	ACE2	−8.18	1.01	His374, His378, Glu402

a BE, binding energy.

b H-bond, hydrogen bond.

c Positive control.

d Positive control.

e Positive control.

f Positive control.

g Positive control.

h Positive control.

Ala, alanine; Asp, aspartic acid; Arg, arginine; Gln, glutamine; Glu, glutamic acid; Gly, glycine; His, histidine; Lys, lysine; Met, methionine; Ser, serine; Thr, threonine; Tyr, tyrosine.

As shown in Table 3, diphyllin exhibited higher affinity to Topo I with the binding energy (BE) of -6.72 kcal/mol, and the theoretical IC_50_ value of 11.95 μM, lower than the positive control 5-FU (−3.70 kcal/mol and 1.95 mM), and slightly higher than Topo I inhibitor camptothecin (−7.57 kcal/mol and 2.85 μM). Kaempferol 3-*O*-glucoside displayed lower affinity to Topo I, and the BE as well as its theoretical IC_50_ value, was computed as −3.59 kcal/mol and 2.35 mM, higher than 5-FU, camptothecin, and diphyllin. With regard to Topo II, podophyllotoxin showed a strong affinity, which its BE and the theoretical IC_50_ value were calculated as −9.45 kcal/mol and 117.98 nM, lower than Topo II inhibitor etoposide (−7.62 kcal/mol and 2.59 μM), and kaempferol 3-*O*-glucoside (−5.28 kcal/mol and 134.95 μM). As for COX-2, the data of docking simulation unveiled that diphyllin expressed a high binding affinity value of −9.95 kcal/mol, and the theoretical IC_50_ value was 51.12 nM, which was relatively lower than the positive control indomethacin (−9.18 kcal/mol and 186.44 nM). Furthermore, diphyllin *O*-glucoside manifested a relatively low affinity to COX-2 with the BE of -4.4 kcal/mol and the theoretical IC_50_ value of 568.94 μM, higher than diphyllin. With respect to ACE2, diphyllin was discovered with a high binding affinity of −7.07 kcal/mol and the theoretical IC_50_ value at 6.52 μM, whereas chloroquine (as the positive control) and MLN-4760 (as the ACE2 inhibitor) were 1.01 and 738.62 μM with the BE of −8.18 and −4.27 kcal/mol, respectively. Similarly, diphyllin *O*-glucoside exerted a relatively strong binding efficiency and indicated the BE of −6.59 kcal/mol with ACE2 from the docking process with the theoretical IC_50_ value at 14.87 μM, respectively. Most importantly, several bioactive ligands screened and identified with the larger EF values possessed lower binding energies and inhibitory effects compared with the same group by molecular docking analysis based on ultrafiltration screening and other *in vitro* bioactivity assays. For instance, diphyllin with large EF values exerted high affinities to Topo I and COX-2, possessing antiproliferative and anti-inflammatory effects confirmed by *in vitro* bioactivity assays and molecular docking analysis. Podophyllotoxin with a relatively large EF value displayed a good affinity to Topo II, which indicated this compound was provided with good antiproliferative activity. The molecular docking results were in full compliance with the ultrafiltration screening and *in vitro* bioactivity verification results, thus further validating the feasibility of the molecular docking method.

## Discussion

### Screening for Topo I, Topo II, COX-2, and ACE2 Ligands in *P. sinense*

Generally, the conventional strategy for the characterization of bioactive components from the medicinal plant is to extract, separate, and identify its chemical constituents by employing traditional phytochemical methods, and then to conduct various biological activity tests combined with pharmacological models to screen each chemical compound obtained (Zhang et al., 2013; Wang et al., 2019). However, the entire operation process was very time-consuming and labor-intensive. In addition, due to the inherent chemical complexity of herbal medicine and its diversified action targets of natural medicines corresponding to a wide range of medicinal uses for various disease indications, the traditional screening method cannot fully reflect the chemical basis of its medicinal effects (Wang et al., 2019).

**FIGURE 6:**
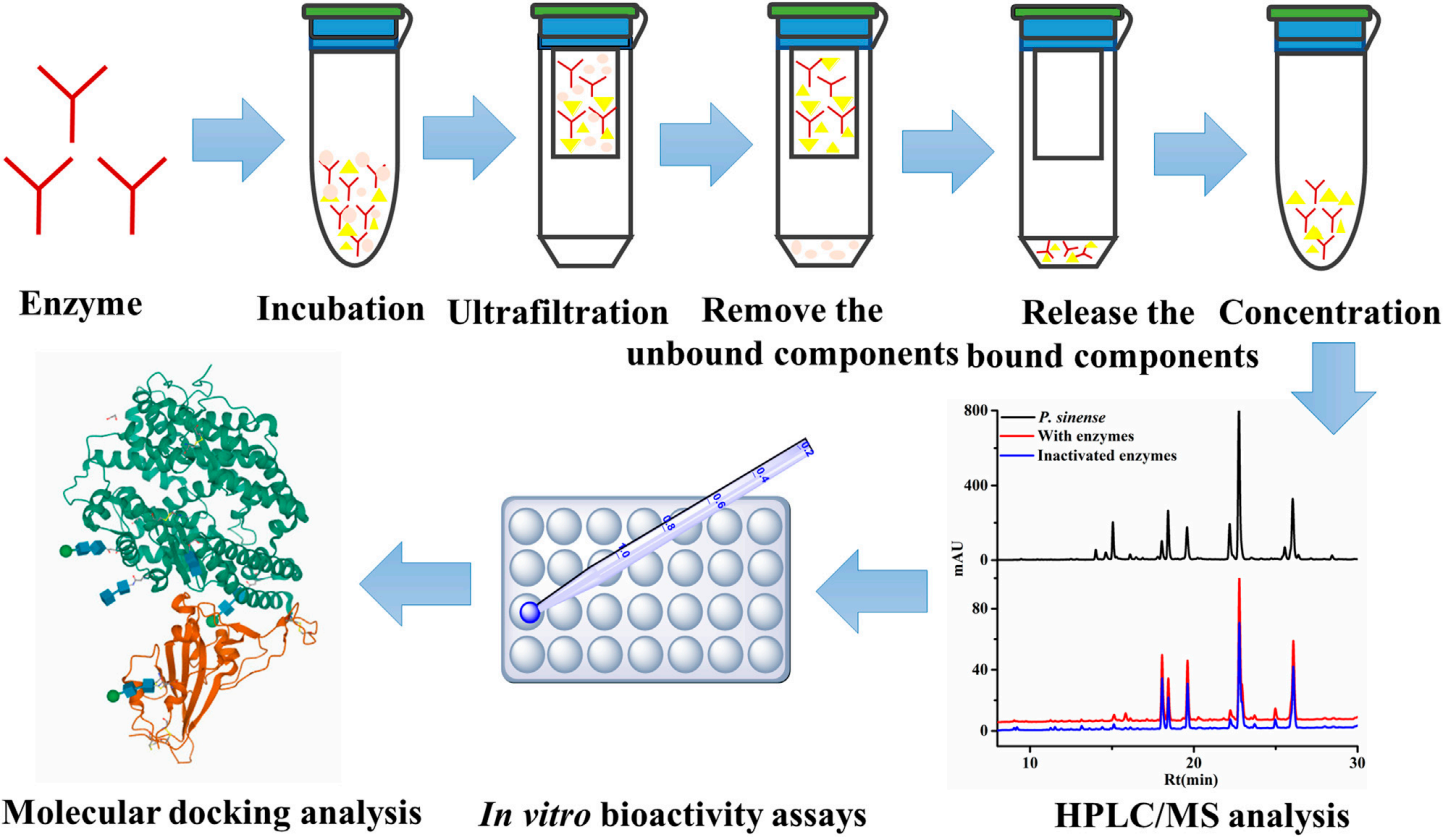
Schematic diagram of UF–HPLC/MS assay to screen and identify the ligands against different drug targets.

As a consequence, in order to address the above limitations and challenges, biological activity-oriented research on the interactions between target enzymes and the small active molecules integrated with the UF–LC/MS approach may provide a good solution. A schematic diagram of the UF–LC/MS screening assay is illustrated in Figure 6. Compared with conventional phytochemical methods, such as thin-layer chromatography (TLC), repeated column chromatography, and low throughput screening tests with purified individual compounds, this strategy can not only rapidly screen out the potential active components against specific targets for certain diseases from crude mixtures without tedious separation process, but also contribute partly to the elaboration of the mechanisms of action for the traditional herbal medicine of interest at the cellular and molecular levels; thereby would offer new tactics for the better use or improving the therapeutic efficacy of diseases (Qin et al., 2015). In light of that, the UF–LC/MS approach was applied to rapidly screen and identify 7, 10, 6, and 7 potential active ligands against Topo I, Topo II, COX-2, and ACE2 from *P. sinense*, respectively.

**FIGURE 7:**
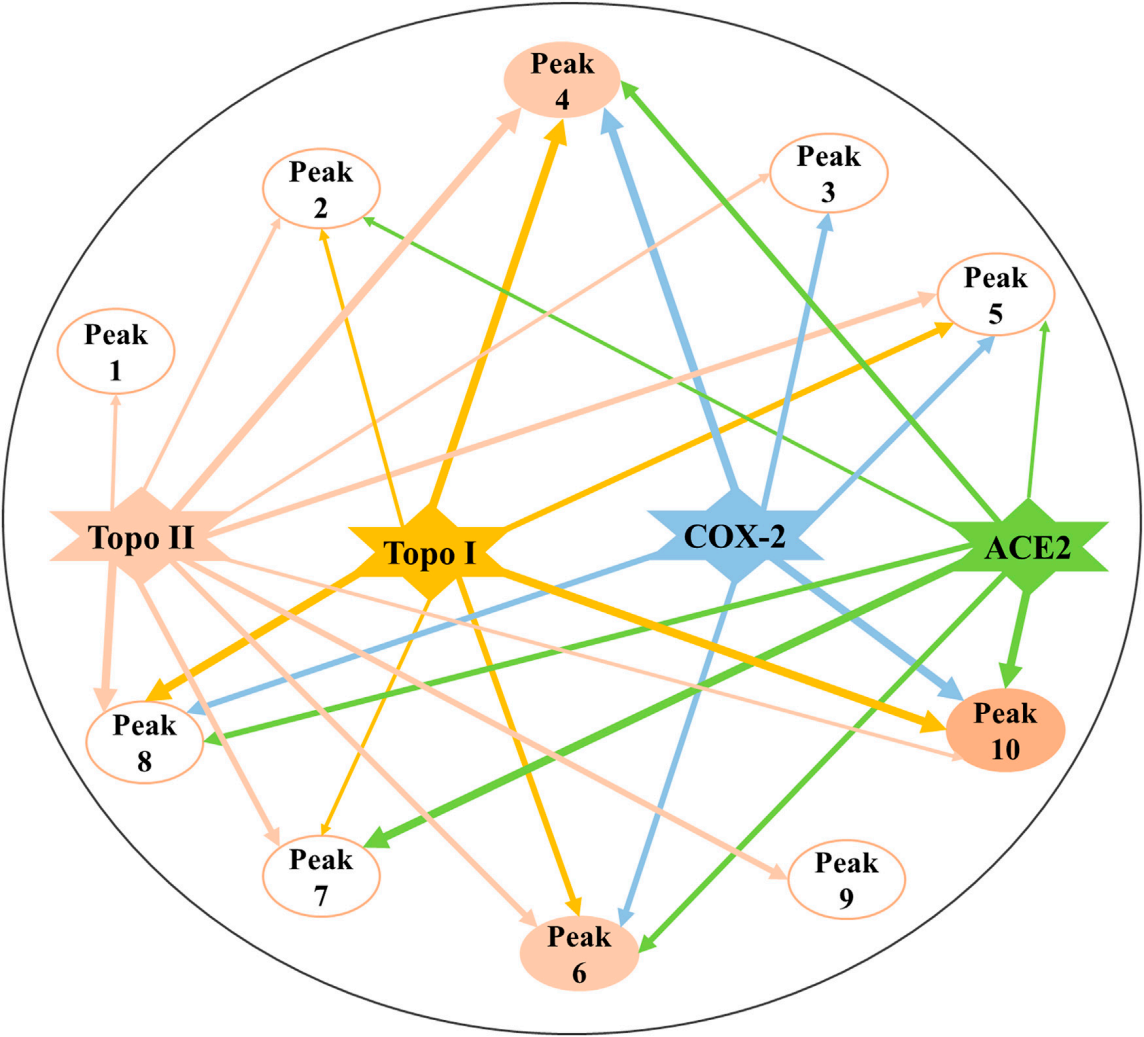
Constructed multi-component and multi-target network incorporating the potential bioactive components screened out with multiple drug targets. The line thicknesses roughly represent the binding or action intensity for their corresponding interactions.

According to the EF values of each component screened from the crude extracts of *P. sinense*, peaks 4, 6, 8, and 10 exhibited relatively higher affinity with Topo I or Topo II, and they were preliminarily inferred to be primary antiproliferative active compounds; in addition, peak 10 exerted stronger binding affinity with COX-2 compared with other peaks, and it was presumed to be the dominating anti-inflammatory active component; and there are several compounds, such as peaks 4, 6, 7, 8, and 10, revealing better binding affinities to the ACE2, which may be its prime antiviral active ingredients. On the one hand, peak 8 associated with Topo I and Topo II exhibited relatively good affinity, which was speculated that this composition may act on these three enzymes so as to exert potential antitumor effects. And the same components like peak 4 binding to Topo I, Topo II, COX-2, and ACE2 exerted relatively good affinity. Similarly, peak 10 presented good affinity to Topo I, COX-2, and ACE2. These results indicated that there existed in the alike bioactive components in *P. sinense* for the multipurpose pharmacological effects. On the other hand, the compounds with larger EF values were more strongly bound to the target enzyme, and more ingredients can be retained during their reactions with the drug targets. In the meantime, due to a large number of active components screened out, there existed synergistic effects among these compounds, jointly exerting multiple effects in clinical practice. Theoretically, the discrepant EF values may be ascribed to their competitively differential interactions with Topo I, Topo II, COX-2, and ACE2. In order to further explore the mechanisms of action underlying the multifunctional use of *P. sinense* in the multi-component and multi-target manner, the potential bioactive compounds screened out from *P. sinense* with multiple drug targets including Topo I, Topo II, COX-2 and ACE2 were then reconstructed as interaction network diagrams to better demonstrate the correlations between the diverse chemical components and their corresponding targets, as shown in Figure 7.

### *In vitro* Bioactive Validation of the Representative Bioactive Compounds

Considering the above screening results listed in Table 1, lignans accounted for the primary bioactive constituents, which was consistent with the results revealed by the previous phytochemical studies (Ma et al., 1993). Furthermore, lignans also exerted various and striking pharmacological activities, including antiproliferative, anti-inflammatory, antiviral and insecticidal effects (Yuan et al., 2011; Liu et al., 2015). Subsequently, further functional assays were carried out to validate their activities so as to better correlate the phytochemical components with pharmacological activities. For the underlying antiproliferative effects, diphyllin and podophyllotoxin with the higher specific binding to Topo I or Topo II in view of our screening experiments and the verification results reported in the literature, we preliminarily inferred that these compounds were considered as the potential Topo I or Topo II ligands and possessed remarkable anti-tumor activity. It was assumed that these components could exert cytotoxic activity on multifarious cancer cell lines by interfering with cell cycle progression, giving rise to DNA damage and inducing apoptosis. As shown in Figure 4, it could be observed that the inhibitory rates of these selected compounds were consistent with the results of ultrafiltration screening based on their EF values. Among these verified components, diphyllin and podophyllotoxin with larger EF values displayed good inhibitory effects on A549 and HT-29 cells compared with the positive controls of 5-FU and etoposide for antiproliferative assay *in vitro*. Compared with the positive control indomethacin, podophyllotoxin and diphyllin with higher EF values showed favorable inhibitory activities for COX-2 inhibitory assay *in vitro*. Thus, it could be seen that the above successful *in vitro* verification experiments of bioactive components screened from *P. sinense* comprehensively clarified the feasibility of the UF-LC/MS approach. Meanwhile, this technology can be employed efficiently to guide further research on the correlation between bioactive constituents and multi-pharmacological activities linked to multiple drug targets, which can provide new opportunities for discovering more therapeutic agents from natural products.

### Molecular Docking Studies

Several component groups had been rapidly screened from the crude extract of *P. sinense* and preliminarily considered to possess potentially promising antiproliferative, anti-inflammatory, and antiviral effects as shown in Table 1. Therein, diphyllin displayed a high affinity to Topo I, COX-2, and ACE2 with large EF values of 4.41, 5.25, and 3.68, respectively. Diphyllin *O*-glucoside exhibited relatively low and moderate affinity to COX-2 and ACE2 with the EF values of 1.14 and 1.37 compared with the corresponding drug targets in the same group, respectively. Podophyllotoxin with a relatively large EF value of 1.17 exerted a good affinity to Topo II. Kaempferol 3-*O*-glucoside with the EF values of 0.37 and 0.18 indicated a relatively low affinity to Topo I and Topo II, respectively. The molecular docking simulation exhibited that these bioactive ligands screened were chiefly docked within the active sites of four drug targets of interest. Meanwhile, the docking analysis could further elaborate the interactions and the underlying mechanisms of action between these potentially bioactive compounds and multiple drug targets including Topo I, Topo II, COX-2, and ACE2.

The docking results verified that the chemical structures of the bioactive ligands significantly affected the interaction sites and binding affinities with Topo I, Topo II, COX-2, and ACE2. More importantly, the active sites and hydrogen bonds (H-bonds) played very crucial roles in the catalytic ability of Topo I, Topo II, COX-2, and ACE2. When several compounds and their respective positive controls were docked with Topo I, Topo II, COX-2, and ACE2, the BE and the theoretical IC_50_ values were ordered as camptothecin > diphyllin > 5-FU; podophyllotoxin > etoposide; diphyllin > indomethacin; chloroquine > diphyllin > diphyllin *O*-glucoside > MLN-4760; the amount of formed H-bonds was ranked as follows: diphyllin = camptothecin > 5-FU; podophyllotoxin > etoposide; diphyllin > indomethacin; and diphyllin *O*-glucoside > chloroquine = MLN-4760 > diphyllin. The results indicated that these compounds exerted good inhibitory activities against Topo I, Topo II, COX-2 and ACE2.

Among these compounds, diphyllin was lodged into the active pocket of the Topo I-DNA complex by forming H-bonds between the hydroxyl groups of B ring and the key amino acid residue Asp533. In the meantime, H-bonds with DNA residue Dc112 enhanced their interaction to DNA strands. Furthermore, the lactone ring 5-oxo of its C ring shaped H-bonds with the residues Arg364 and Da113. In addition, it could also be clearly found that this top active ligand was provided with a strong binding with COX-2. The lactone ring 5-oxo group of C ring of diphyllin interacted with the key residues of COX-2 including Arg120 and Tyr355 (Kurumbail et al., 1996). Moreover, it built one H-bond between the hydroxyl groups of B ring and the residue Ala527 to enhance the binding forces. It also formed one H-bond between the oxygen-containing groups of the methylenedioxybenzene ring and the residue His90. Additionally, the side chains of Val349 and Val523 amino acid residues displayed Pi-Alkyl and Pi-sigma forces to its B ring, while on the other side, some residues such as Ala516, Gly526, Tyr385 and so forth surrounded the compound through van der Waals’ forces. There were also some reports stating that diphyllin inhibited the activity of vacuolar-type H^+^-ATPase (V-ATPase) with the effective test concentration (EC) of 0.3 μM using cell-based assays targeting the viral entry previously (Prasansuklab et al., 2021). In the present study, diphyllin with a high binding affinity to ACE2 was shortlisted as prominent ligands by applying molecular docking analysis. It was observed that the lactone ring of diphyllin formed one H-bond with the amino acid residue Asp382. Meanwhile, the methoxybenzene groups of A ring of this compound also shaped one H-bond with the residue Asp350 on ACE2 to further enhance the affinity. The key amino acid residues His378 and His401 exhibited Pi-Pi T-shaped force to the phenyl-containing groups of the methylenedioxybenzene ring and the hydroxyl groups of B ring of this compound. Likewise, the lactone ring 5-oxo group of C ring of diphyllin *O*-glucoside formed H-bonds with the residue Ala348. Moreover, the residues Arg514 and Asn394 built H-bonds to the methoxybenzene groups of the A ring and the oxygen-containing groups of the methylenedioxybenzene ring of this compound, respectively. Furthermore, the hydroxyl group of the arabinosyl moiety of this compound formed three stable H-bonds with different types of amino acid residues His378, Glu375, Pro346 attached to ACE2. Additionally, the residues Glu402 and His401 shared Pi-Sigma and Pi-Lone Pair forces to the phenyl groups of A and D rings of this compound. The docking result was similar to chloroquine and MLN-4760 (as the positive controls) which formed several stable H-bonds such as His374, His378 or Glu375, indicating these H-bonds formed with residues on ACE2 played a pivotal role in the targeted binding process. With regard to Topo II, the trimethoxyphenyl group of E ring of podophyllotoxin shared H-bonds with the residue Gln778, serving as an H-donor to the ring. At the same time, podophyllotoxin intercalated into the DNA complex through H-bonds with the residues Da12 and Dt9. It was observed to form three conventional H-bonds with the residues Da12, Dt9, and Gln778, of which interacted with Topo II inhibitor etoposide as well. Moreover, there were related literature reports that podophyllotoxin could be applied as a cell division inhibitor possessing antitumor activity in the metaphase of cell division, which inhibited the polymerization of microtubules and mitosis of the nucleus, and thus was suspended in the metaphase (Liu et al., 2015). Furthermore, there existed other essential driven forces such as the Van der Waals force, hydrophobic and electrostatic effects between those potential active ingredients and Topo I, Topo II, COX-2, or ACE2 in the process of molecular docking simulation. Above all, the molecular docking analysis was consistent with the results of affinity ultrafiltration screening. Therefore, these components were promising to be developed as potential antiproliferative, anti-inflammatory, and antiviral lead candidates for the discovery of target-specific therapeutic drugs.

## Conclusion

The therapeutic effects of medicinal plants are mainly on account of the integral complex chemical properties of multiple secondary metabolites, which could interact on multiple drug targets or synergistically interact with various biochemical pathways to conduct its holistic curative effects. This study aimed to comprehensively explore the underlying pharmacological mechanisms of action for *P. sinense* in a multi-target and multi-component way using Topo I, Topo II, COX-2, and ACE2 as potential target enzymes. Besides, combined these drug targets with the UF–LC/MS screening strategy, we were able to quickly screen out multi-functional components responsible for the antiproliferative, anti-inflammatory, and antiviral activities from the crude extracts of *P. sinense* for the first time. In total, 7, 10, 6, and 7 components were fished out as the potential Topo I, Topo II, COX-2, and ACE2 ligands, respectively. It is obviously that these components could act synergistically on the corresponding drug targets in a multi-component manner to take their effects. On the other side, among these active components, the individual compound that bound well to diverse target enzymes, such as Topo I and Topo II, had been detected as the single bioactive ingredient in *P. sinense* to simultaneously exert its intricate action mechanisms in a multi-target way. The activity verification of representative components, such as diphyllin and podophyllotoxin, further confirmed the feasibility of our screening method, and their potential pharmacological effects with *in vitro* assays. Meanwhile, the molecular docking simulation further indicated and validated the inhibitory mechanisms between Topo I, Topo II, COX-2 or ACE2 and those representative constituents such as diphyllin, podophyllotoxin and diphyllin *O*-glucoside. To the best of our knowledge, this is the first report to systematically investigate the antiproliferative, anti-inflammatory and antiviral activities, their potentially responsible chemical components and mechanisms by employing bio-affinity ultrafiltration with the multiple drug targets combined with LC/MS. This work will not only facilitate and elucidate the correlations between potential bioactive components and their biological targets including the underlying mechanisms of action, but also lay a further solid foundation for discovering more novel and curative agents from *P. sinense* or other medicinal plants.

## Data Availability

The raw data supporting the conclusion of this article will be made available by the authors, without undue reservation.
